# Supplementary material for: Targeting earlier diagnosis: What symptoms come first in Degenerative Cervical Myelopathy?
Source: PLoS One. 2023 Mar 31;18(3):e0281856. doi: 10.1371/journal.pone.0281856 (PMC10065274; doi:10.1371/journal.pone.0281856)
Supplement: S1 Appendix — (DOCX) [file pone.0281856.s001.docx]

**S1 Appendix. Copy of round 2 internet survey.**

1. Do you suffer from any of the conditions encompassed by the term Degenerative Cervical Myelopathy*? (*These include: Cervical Spondylotic Myelopathy (CSM), Ossification of the Ligamentum Flavum (OLF), Ossification of the Posterior Longitudinal Ligament (OPLL) and Degenerative Disc Disease (DDD).)
   - Yes
   - No, but I know someone who does
   - No
2. What is your age?
   - In years:
3. What gender are you?
   - Male
   - Female
4. How long have you suffered with DCM?
   - In years:
   - and months:
5. How long did it take to be diagnosed with DCM?
   - In years:
   - and months:
6. Have you had surgery for DCM?
   - Yes
   - No
7. What term do you think best describes those who provide assistance to DCM sufferers?
   - Supporters
   - Carers
   - Helpers
   - Care givers
   - Friends
   - Other (please specify)
8. mJOA: With regards to the function of your hands?
   - I am unable to move my hands at all
   - I am not able to eat with a spoon, but I can move my hands
   - I am not able to do up my shirt buttons, but I can eat with a spoon
   - I am able to button my shirt, but it is extremely difficult
   - My hands are unaffected
9. mJOA: With regards to your legs?
   - I am unable to feel or move my legs
   - I can feel my legs, but I cannot move them
   - I am able to move my legs but I cannot walk
   - I can walk, but I require a walking aid and only on the flat
   - I can walk up and down stairs, but I must hold onto the handrail
   - I can walk up and down stairs without holding onto the handrail
   - I can walk unaided, with only a mild instability
   - My walking is unaffected
10. mJOA: With regards to the feeling in your hands?
    - I have no feeling in my hands
    - I have significant loss of feeling (incl. numbness, tingling) or pain in my hands
    - I have mild loss of feeling (incl. numbness, tingling) in my hands
    - I have normal feeling in my hands
11. mJOA: With regards to your bladder function?
    - I am unable to urinate voluntarily
    - I have marked urination difficulties
    - I have mild urination difficulties
    - I have no urination difficulties
12. What symptoms do you experience from DCM? For each symptom, tick the first box if you currently have the symptom and tick the second box if it was one of your first symptoms.
    - Anxiety
    - Arm pain
    - Arm stiffness
    - Back pain
    - Clumsiness
    - Depression/low mood
    - Difficulty breathing when lying flat
    - Difficulty breathing when performing physical activity
    - Difficulty emptying bladder
    - Dragging legs
    - Erectile Dysfunction
    - Faecal incontinence
    - Falls
    - Fatigue
    - Hand shaking
    - Heavy legs
    - Hot flushes and/or sweating
    - Impaired cognition
    - Insomnia
    - Lack of control of legs
    - Leg pain
    - Leg shaking
    - Leg stiffness
    - Muscle spasms or twitches (in your arms)
    - Muscle spasms or twitches (in your legs)
    - Neck clicking
    - Neck pain
    - Neck stiffness
    - Numbness
    - Pins and needles
    - Reduced dexterity (less able to perform complex tasks with your hands)
    - Reduced grip strength
    - Symptom variability day by day
    - Symptom variability hour by hour
    - Urinary incontinence
    - Waking to go to the toilet
13. If you suffer from any symptoms, caused by DCM, that have not been mentioned above, please enter them here:
14. How does DCM affect your life? For each potential effect on your life, tick the first box if it currently affects you and tick the second box if it was one of the first things that affected you.
    - Muscle weakness
    - Reduced activity such that you are unable to have fun
    - Reduced walking distance
    - Difficulties climbing stairs
    - Unable to get comfortable in bed
    - Difficulty with travel
    - Difficulty with planning life
    - Unable to exercise
    - Reduced activity such that you are unable to work
    - Financial difficulties
    - Difficulties with social interaction
    - Falls
    - Inability to turn over in bed
    - Unable to drive
    - Unable to get up out of a chair
    - Unable to get out of bed
15. If there are any effects on your life, caused by DCM, that have not been mentioned above, please enter them here:
16. Arm/Leg Function: How much do these problems affect you?
    - 1. Not at all
    - 2. A little
    - 3. Moderately
    - 4. Quite a bit
    - 5. Extremely
17. Arm/Leg Function: How much do your problems affect those who support you?
    - 1. Not at all
    - 2. A little
    - 3. Moderately
    - 4. Quite a bit
    - 5. Extremely
18. Immobility: How much do these problems affect you?
    - 1. Not at all
    - 2. A little
    - 3. Moderately
    - 4. Quite a bit
    - 5. Extremely
19. Immobility: How much do your problems affect those who support you?
    - 1. Not at all
    - 2. A little
    - 3. Moderately
    - 4. Quite a bit
    - 5. Extremely
20. Genitourinary Issues: How much do these problems affect you?
    - 1. Not at all
    - 2. A little
    - 3. Moderately
    - 4. Quite a bit
    - 5. Extremely
21. Genitourinary Issues: How much do your problems affect those who support you?
    - 1. Not at all
    - 2. A little
    - 3. Moderately
    - 4. Quite a bit
    - 5. Extremely
22. Sleep: How much do these problems affect you?
    - 1. Not at all
    - 2. A little
    - 3. Moderately
    - 4. Quite a bit
    - 5. Extremely
23. Sleep: How much do your problems affect those who support you?
    - 1. Not at all
    - 2. A little
    - 3. Moderately
    - 4. Quite a bit
    - 5. Extremely
24. Sensation: How much do these problems affect you?
    - 1. Not at all
    - 2. A little
    - 3. Moderately
    - 4. Quite a bit
    - 5. Extremely
25. Sensation: How much do your problems affect those who support you?
    - 1. Not at all
    - 2. A little
    - 3. Moderately
    - 4. Quite a bit
    - 5. Extremely
26. Breathing: How much do these problems affect you?
    - 1. Not at all
    - 2. A little
    - 3. Moderately
    - 4. Quite a bit
    - 5. Extremely
27. Breathing: How much do your problems affect those who support you?
    - 1. Not at all
    - 2. A little
    - 3. Moderately
    - 4. Quite a bit
    - 5. Extremely
28. Pain: How much do these problems affect you?
    - 1. Not at all
    - 2. A little
    - 3. Moderately
    - 4. Quite a bit
    - 5. Extremely
29. Pain: How much do your problems affect those who support you?
    - 1. Not at all
    - 2. A little
    - 3. Moderately
    - 4. Quite a bit
    - 5. Extremely
30. Psychosocial: How much do these problems affect you?
    - 1. Not at all
    - 2. A little
    - 3. Moderately
    - 4. Quite a bit
    - 5. Extremely
31. Psychosocial: How much do your problems affect those who support you?
    - 1. Not at all
    - 2. A little
    - 3. Moderately
    - 4. Quite a bit
    - 5. Extremely
32. Disease Variability: How much do these problems affect you?
    - 1. Not at all
    - 2. A little
    - 3. Moderately
    - 4. Quite a bit
    - 5. Extremely
33. Disease Variability: How much do your problems affect those who support you?
    - 1. Not at all
    - 2. A little
    - 3. Moderately
    - 4. Quite a bit
    - 5. Extremely
34. Please could you rank the following categories in order of their impact on you:(with 1 having the greatest impact and 9 having the least impact)
    - Arm/Leg Function
    - Immobility
    - Genitourinary Issues
    - Sleep
    - Sensation
    - Breathing
    - Pain
    - Psychosocial
    - Disease Variability
