# Supplementary material for: Targeting earlier diagnosis: What symptoms come first in Degenerative Cervical Myelopathy?
Source: PLoS One. 2023 Mar 31;18(3):e0281856. doi: 10.1371/journal.pone.0281856 (PMC10065274; doi:10.1371/journal.pone.0281856)
Supplement: S2 Appendix — (DOCX) [file pone.0281856.s002.docx]

**S2 Appendix. Copy of round 3 internet survey.**

1. Do you suffer from myelopathy (disease of the spinal cord)?
   - Yes
   - No
2. Is your myelopathy caused by any of the conditions encompassed by the term Degenerative Cervical Myelopathy*? (*These include: Cervical Spondylotic Myelopathy (CSM), Ossification of the Ligamentum Flavum (OLF), Ossification of the Posterior Longitudinal Ligament (OPLL) and Degenerative Disc Disease (DDD).)
   - Yes
   - No
3. What is your age?
   - In years:
4. What gender are you?
   - Male
   - Female
5. How long have you suffered with DCM?
   - In years:
   - and months:
6. How long did it take to be diagnosed with DCM?
   - In years:
   - and months:
7. Have you had surgery for DCM?
   - Yes
   - No
8. With regards to the function of your hands?
   - I am unable to move my hands at all
   - I am not able to eat with a spoon, but I can move my hands
   - I am not able to do up my shirt buttons, but I can eat with a spoon
   - I am able to button my shirt, but it is extremely difficult
   - My hands are unaffected
9. With regards to your legs?
   - I am unable to feel or move my legs
   - I can feel my legs, but I cannot move them
   - I am able to move my legs but I cannot walk
   - I can walk, but I require a walking aid and only on the flat
   - I can walk up and down stairs, but I must hold onto the handrail
   - I can walk up and down stairs without holding onto the handrail
   - I can walk unaided, with only a mild instability
   - My walking is unaffected
10. With regards to the feeling in your hands?
    - I have no feeling in my hands
    - I have significant loss of feeling (incl. numbness, tingling) or pain in my hands
    - I have mild loss of feeling (incl. numbness, tingling) in my hands
    - I have normal feeling in my hands
11. With regards to your bladder function?
    - I am unable to urinate voluntarily
    - I have marked urination difficulties
    - I have mild urination difficulties
    - I have no urination difficulties
12. What symptoms do you currently experience due to DCM?For each symptom, tick the box if you currently experience the symptom as a result of DCM.
    - Hand shaking
    - Reduced grip strength
    - Reduced dexterity (less able to perform complex tasks with your hands)
    - Muscle spasms or twitches (in your arms)
    - Leg shaking
    - Heavy legs
    - Dragging legs
    - Muscle spasms or twitches (in your legs)
    - Falls
    - Lack of control of legs
    - Clumsiness
    - Difficulty emptying bladder
    - Urinary incontinence
    - Faecal incontinence
    - Erectile Dysfunction
    - Symptom variability day by day
    - Symptom variability hour by hour
    - Insomnia
    - Waking to go to the toilet
    - Difficulty breathing when performing physical activity
    - Difficulty breathing when lying flat
    - Hot flushes and/or sweating
    - Arm numbness
    - Hand numbness
    - Leg numbness
    - Pins and needles in your hand
    - Pins and needles in your arm
    - Pins and needles in your leg
    - Neck pain
    - Arm pain
    - Leg pain
    - Back pain
    - Neck stiffness
    - Arm stiffness
    - Leg stiffness
    - Neck clicking
    - Depression/low mood
    - Anxiety
    - Impaired cognition
    - Fatigue
    - Headache
    - Poor balance
    - Shoulder pain
    - Electric shock-like sensations down your back (Lhermitte's sign)
    - Dizziness
    - Female sexual dysfunction
    - Altered temperature sensation
    - Pain from previously non-painful sensations (Allodynia)
    - Choking/swallowing problems
    - Face pain
    - Face numbness
    - Constipation
    - Eyesight problems
    - Ringing in your ears (Tinnitus)
    - Abdominal pain
    - Nausea & vomiting
13. Which of these was the first symptom you experienced as a result of DCM?Tick the circle for the first symptom you experienced due to DCM (if you experienced more than one, and cannot recall which came first, please select the most significant one at that time)
    - Hand shaking
    - Reduced grip strength
    - Reduced dexterity (less able to perform complex tasks with your hands)
    - Muscle spasms or twitches (in your arms)
    - Leg shaking
    - Heavy legs
    - Dragging legs
    - Muscle spasms or twitches (in your legs)
    - Falls
    - Lack of control of legs
    - Clumsiness
    - Difficulty emptying bladder
    - Urinary incontinence
    - Faecal incontinence
    - Erectile Dysfunction
    - Symptom variability day by day
    - Symptom variability hour by hour
    - Insomnia
    - Waking to go to the toilet
    - Difficulty breathing when performing physical activity Difficulty breathing when lying flat
    - Hot flushes and/or sweating
    - Arm numbness
    - Hand numbness
    - Leg numbness
    - Pins and needles in your hand
    - Pins and needles in your arm
    - Pins and needles in your leg
    - Neck pain
    - Arm pain
    - Leg pain
    - Back pain
    - Neck stiffness
    - Arm stiffness
    - Leg stiffness
    - Neck clicking
    - Depression/low mood
    - Anxiety
    - Impaired cognition
    - Fatigue
    - Headache
    - Poor balance
    - Shoulder pain
    - Electric shock-like sensations down your back (Lhermitte's sign)
    - Dizziness
    - Female sexual dysfunction
    - Altered temperature sensation
    - Pain from previously non-painful sensations (Allodynia)
    - Choking/swallowing problems
    - Face pain
    - Face numbness
    - Constipation
    - Eyesight problems
    - Ringing in your ears (Tinnitus)
    - Abdominal pain
    - Nausea & vomiting
14. Have you ever had a confirmed diagnosis of Fibromyalgia?
    - Yes
    - No
15. How does DCM affect your life? For each potential effect on your life, tick the box if it currently affects you.
    - Falls
    - Unable to drive
    - Unable to exercise
    - Difficulties climbing stairs
    - Unable to get up out of a chair
    - Unable to get out of bed
    - Muscle weakness
    - Reduced walking distance
    - Reduced activity such that you are unable to have fun Reduced activity such that you are unable to work
    - Difficulty with travel
    - Difficulty with planning life
    - Inability to turn over in bed
    - Unable to get comfortable in bed
    - Difficulties with social interaction
    - Financial difficulties
    - Difficulty parenting and in family life
    - Lifting heavy objects is difficult/painful
    - Reduced sex life
    - Difficulty thinking/concentrating/memory problems
    - Difficulty in performing household tasks
    - Living in fear of damaging spinal cord further from falls/trauma
16. How did DCM first affect your life? Tick the circle for the first affect on your life (if you experienced more than one, and cannot recall which came first, please select the most significant one at that time).
    - Falls
    - Unable to drive
    - Unable to exercise
    - Difficulties climbing stairs
    - Unable to get up out of a chair
    - Unable to get out of bed
    - Muscle weakness
    - Reduced walking distance
    - Reduced activity such that you are unable to have fun Reduced activity such that you are unable to work
    - Difficulty with travel
    - Difficulty with planning life
    - Inability to turn over in bed
    - Unable to get comfortable in bed
    - Difficulties with social interaction
    - Financial difficulties
    - Difficulty parenting and in family life
    - Lifting heavy objects is difficult/painful
    - Reduced sex life
    - Difficulty thinking/concentrating/memory problems
    - Difficulty in performing household tasks
    - Living in fear of damaging spinal cord further from falls/trauma
17. Do you think the above categories adequately describe the different ways in which DCM can affect people?
    - Yes
    - No (please give a reason why)
