# Supplementary material for: Targeting earlier diagnosis: What symptoms come first in Degenerative Cervical Myelopathy?
Source: PLoS One. 2023 Mar 31;18(3):e0281856. doi: 10.1371/journal.pone.0281856 (PMC10065274; doi:10.1371/journal.pone.0281856)
Supplement: S1 Table — *Outcomes added after respondent suggestion in round 2. 2 shortlisted symptoms (italicised) were expanded “anatomically” for the longlist. (DOCX) [file pone.0281856.s003.docx]

**S1 Table. Short and longlisted survey outcomes.**

| **Round 2 Survey - Shortlisted Outcomes** | **Round 3 Survey - Longlisted Outcomes** |
| --- | --- |
| Hand shaking | Hand shaking |
| Reduced grip strength | Reduced grip strength |
| Reduced dexterity (less able to perform complex tasks with your hands) | Reduced dexterity (less able to perform complex tasks with your hands) |
| Muscle spasms or twitches (in your arms) | Muscle spasms or twitches (in your arms) |
| Leg shaking | Leg shaking |
| Heavy legs | Heavy legs |
| Dragging legs | Dragging legs |
| Muscle spasms or twitches (in your legs) | Muscle spasms or twitches (in your legs) |
| Falls | Falls |
| Lack of control of legs | Lack of control of legs |
| Clumsiness | Clumsiness |
| Difficulty emptying bladder | Difficulty emptying bladder |
| Urinary incontinence | Urinary incontinence |
| Faecal incontinence | Faecal incontinence |
| Erectile Dysfunction | Erectile Dysfunction |
| Symptom variability day by day | Symptom variability day by day |
| Symptom variability hour by hour | Symptom variability hour by hour |
| Insomnia | Insomnia |
| Waking to go to the toilet | Waking to go to the toilet |
| Difficulty breathing when performing physical activity | Difficulty breathing when performing physical activity |
| Difficulty breathing when lying flat | Difficulty breathing when lying flat |
| Hot flushes and/or sweating | Hot flushes and/or sweating |
| *Numbness* | *Arm numbness* |
|  | *Hand numbness* |
|  | *Leg numbness* |
| *Pins and needles* | *Pins and needles in your hand* |
|  | *Pins and needles in your arm* |
|  | *Pins and needles in your leg* |
| Neck pain | Neck pain |
| Arm pain | Arm pain |
| Leg pain | Leg pain |
| Back pain | Back pain |
| Neck stiffness | Neck stiffness |
| Arm stiffness | Arm stiffness |
| Leg stiffness | Leg stiffness |
| Neck clicking | Neck clicking |
| Depression/low mood | Depression/low mood |
| Anxiety | Anxiety |
| Impaired cognition | Impaired cognition |
| Fatigue | Fatigue |
|  | Headache* |
|  | Poor balance* |
|  | Shoulder pain |
|  | Electric shock-like sensations down your back (Lhermitte's sign)* |
|  | Dizziness* |
|  | Female sexual dysfunction* |
|  | Altered temperature sensation* |
|  | Pain from previously non-painful sensations (Allodynia)* |
|  | Choking/swallowing problems* |
|  | Face pain* |
|  | Face numbness* |
|  | Constipation* |
|  | Eyesight problems* |
|  | Ringing in your ears (Tinnitus)* |
|  | Abdominal pain* |
|  | Nausea & vomiting* |

*Outcomes added after respondent suggestion in round 2. 2 shortlisted symptoms (italicised) were expanded “anatomically” for the longlist.
