# Supplementary material for: Targeting earlier diagnosis: What symptoms come first in Degenerative Cervical Myelopathy?
Source: PLoS One. 2023 Mar 31;18(3):e0281856. doi: 10.1371/journal.pone.0281856 (PMC10065274; doi:10.1371/journal.pone.0281856)
Supplement: S2 Table — Aside from gender and time to diagnosis, the cohorts are closely matched. (DOCX) [file pone.0281856.s004.docx]

**S2 Table. Comparison of demographics between survey cohort and AO Spine observational study.**

| **Demographic parameters, mean ± SD:** | **This study (n=171)** | **Tetreault et al. 2018 (n=679)** |
| --- | --- | --- |
| Age (years) | 53.9 ± 9.5 | 56.4 ± 11.8 |
| mJOA | 11.5 ± 2.8 | 12.5 ± 2.7 |
| Gender (% of males) | 26.3 | 62.4 |
| % Diagnosed/operated late (>6m) | 79.5 | 77.5 |
| Time to diagnosis/surgery (months) | 46.4 ± 68.6 | 26.1 ± 36.4 |

Aside from gender and time to diagnosis, the cohorts are closely matched.
