# Supplementary material for: Targeting earlier diagnosis: What symptoms come first in Degenerative Cervical Myelopathy?
Source: PLoS One. 2023 Mar 31;18(3):e0281856. doi: 10.1371/journal.pone.0281856 (PMC10065274; doi:10.1371/journal.pone.0281856)
Supplement: S1 Fig — Comparison of matched symptoms between this study (white) and the AO Spine prospective observational study of sufferers undergoing surgical treatment for DCM (grey). Error bars represent 95% confidence intervals. (DOCX) [file pone.0281856.s005.docx]

**S1 Fig.** **Symptom frequency comparison with Tetreault et al. 2018.** Comparison of matched symptoms between this study (white) and the AO Spine prospective observational study of sufferers undergoing surgical treatment for DCM (grey). Error bars represent 95% confidence intervals.
